# Impact of preoperative anemia, iron-deficiency and inflammation on survival after colorectal surgery—A retrospective cohort study

**DOI:** 10.1371/journal.pone.0269309

**Published:** 2022-07-27

**Authors:** Messina Bath, André Viveiros, Benedikt Schaefer, Sebastian Klein, Lorenz M. Pammer, Sonja Wagner, Andreas Lorenz, Christopher Rugg, Elisabeth Gasser, Marijana Ninkovic, Marlene Panzer, Elke Pertler, Dietmar Fries, Herbert Tilg, Guenter Weiss, Verena Petzer, Dietmar Öfner-Velano, Heinz Zoller

**Affiliations:** 1 Christian Doppler Laboratory on Iron and Phosphate Biology, Medical University of Innsbruck, Innsbruck, Austria; 2 Department of Medicine I, Gastroenterology, Hepatology and Endocrinology, University Hospital & Medical University of Innsbruck, Innsbruck, Austria; 3 Department of Visceral, Transplant and Thoracic Surgery, Center of Operative Medicine, University Hospital & Medical University of Innsbruck, Innsbruck, Austria; 4 Department of Anaesthesiology and Intensive care, University Hospital & Medical University of Innsbruck, Innsbruck, Austria; 5 VASCage – Research Centre on Vascular Ageing and Stroke, Innsbruck, Austria; 6 Department of Medicine II, Infectious Diseases, University Hospital & Medical University of Innsbruck, Innsbruck, Austria; 7 Christian Doppler Laboratory for Iron Metabolism and Anemia Research, Medical University of Innsbruck, Innsbruck, Austria; 8 Department of Medicine V, Hematology and Oncology, Medical University of Innsbruck, Innsbruck, Austria; Medizinische Fakultat der RWTH Aachen, GERMANY

## Abstract

**Background:**

Anemia is present in up to two-thirds of patients undergoing colorectal surgery mainly caused by iron deficiency and inflammation. As anemia is associated with increased risk of perioperative death, diagnosis and treatment of preoperative anemia according to etiology have been recommended.

**Objective:**

The aim of the present study was to assess if the association between anemia and survival in patients undergoing colorectal surgery was determined by the severity of anemia alone or also by anemia etiology.

**Methods:**

To determine the prevalence of anemia and etiology, preoperative hematological parameters, C-reactive protein, ferritin and transferrin saturation were retrospectively assessed and correlated with outcome in a cohort of patients undergoing colorectal surgery between 2005 and 2019 at the University Hospital of Innsbruck. Anemia was defined as hemoglobin <120 g/L in females and <130 g/L in males. The etiology of anemia was classified on the basis of serum iron parameters, as iron deficiency anemia, anemia of inflammation or other anemia etiologies.

**Results:**

Preoperative anemia was present in 54% (1316/2458) of all patients. Anemia was associated with iron deficiency in 45% (134/299) and classified as anemia of inflammation in 32% (97/299) of patients with available serum iron parameters. The etiology of anemia was a strong and independent predictor of survival, where iron deficiency and anemia of inflammation were associated with better postoperative survival than other anemia etiologies. One year survival rates were 84.3%, 77.3% and 69.1% for patients with iron deficiency anemia, anemia of inflammation and other anemia types. Inflammation indicated by high C-reactive protein is a strong negative predictor of overall survival.

**Conclusions:**

Anemia has a high prevalence among patients undergoing colorectal surgery and rational treatment requires early assessment of serum iron parameters and C-reactive protein.

## Introduction

Large observational studies have identified preoperative anemia as a strong negative predictor of survival and associated anemia with increased morbidity after colorectal surgery [1, 2]. Several guidelines recommend preoperative management of anemia, in terms of diagnosis and therapy [3–5]. The most common cause of anemia in patients undergoing colorectal surgery is iron deficiency, affecting over 50% of patients with colorectal cancer [2, 6, 7]. Inflammation is an alternative or additional risk factor for the development of anemia in patients with colorectal diseases [8].

Low mean corpuscular hemoglobin (MCH) and reduced mean corpuscular volume (MCV) are indicative of but not diagnostic for iron deficiency as the underlying etiology of anemia. Common differential diagnoses for hypochromia and microcytosis include thalassemia syndromes and other hemoglobinopathies. Coincidence of iron and vitamin B12 or folate deficiency can obscure hypochromia and microcytosis. Despite these limitations, red cell indices (MCH, MCV) have been suggested to guide perioperative blood management [9]. Diagnosis of iron deficiency is based on low serum ferritin and reduced transferrin saturation (TSAT). Patients with serum ferritin < 30μg/L are considered iron deficient, although more recent studies show a ferritin threshold of 50μg/L indicates iron deficiency [10–12]. To account for the increase in serum ferritin during inflammation, guidelines recommend that patients with ferritin < 100μg/L and TSAT < 20% should also be treated for iron deficiency [10].

The three pillars of patient blood management (PBM) include optimization of erythropoiesis, minimization of blood loss and hemostasis as well as the optimization of the physiological reserve of anemia [13]. In the perioperative setting, optimization of erythropoiesis is mainly based on intravenous iron supplementation and treatment of the underlying cause of iron deficiency [14]. Several studies have shown that preoperative correction of iron deficiency improves outcome [15–17]. Preoperative anemia screening and intravenous iron supplementation therefore represent an important pillar of PBM [18]. A placebo-controlled trial investigating the effect of preoperative iron therapy on mortality in all patients with anemia regardless of the underlying etiology. Although 82% of the study population had iron deficiency as defined in the protocol, this study did not show a survival benefit after preoperative administration of the intravenous iron formulation ferric carboxymaltose, despite the finding IV iron was effective in correcting anemia [19]. Although a recent meta-analysis confirmed this finding, it also highlighted the limitations of this study and uncertainties in this area [20].

Inflammation cannot only obscure the diagnosis of iron deficiency by increasing serum ferritin, but is also a common cause of anemia [21]. Studies have shown that host inflammation is associated with poor survival in patients with colorectal cancer [22]. The present study is carried out to determine if anemia etiology classified as IDA, anemia of inflammation (AI) or ‘other’ anemia etiologies is a predictor of survival.

The present study was carried out to determine the prevalence of anemia and the association between anemia etiology and survival in a large cohort of patients who underwent colorectal surgery. The underlying hypothesis for this study was that anemia etiology is a determinant of patient outcome.

## Materials and methods

**Fig 1:**
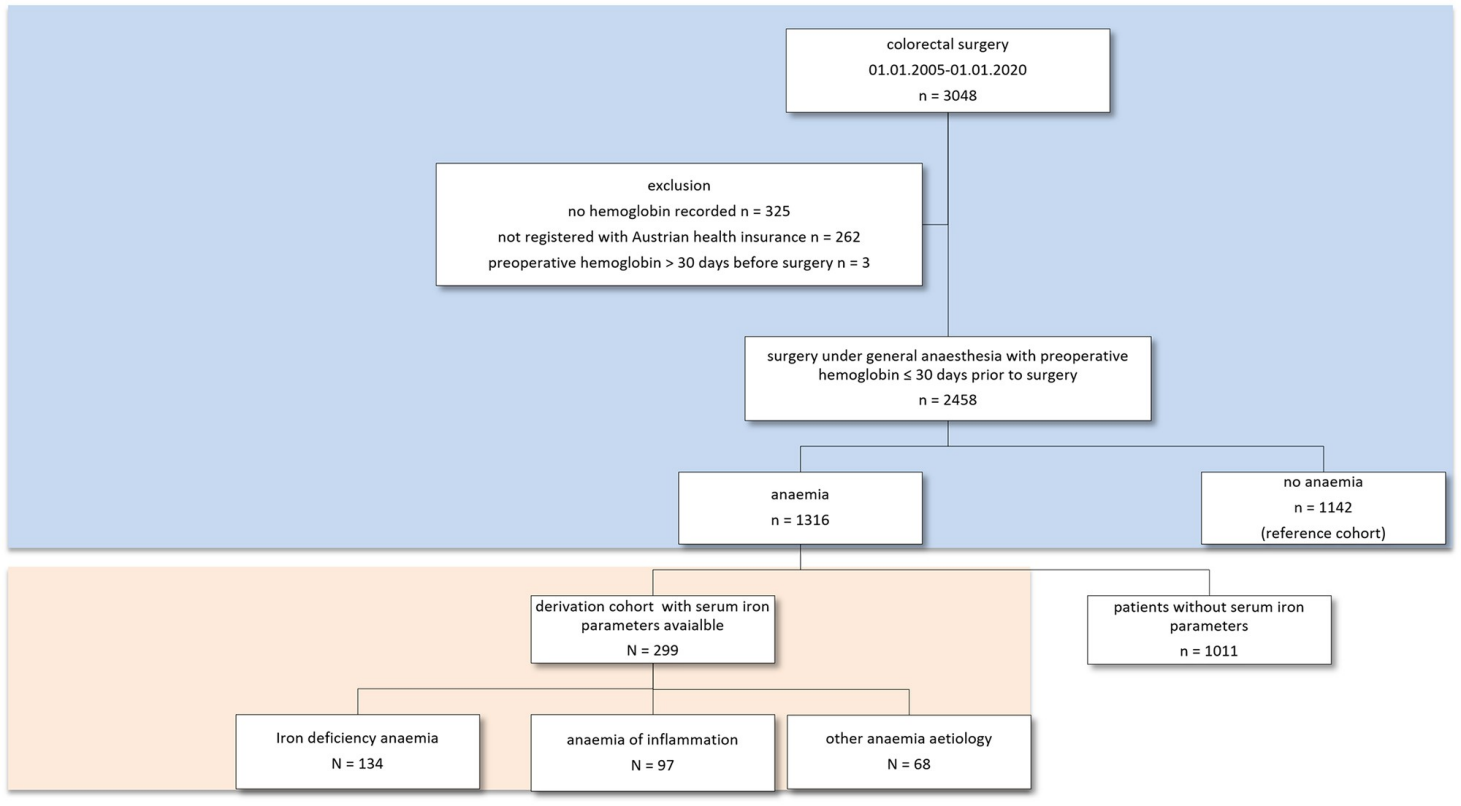
Consort diagram.

This retrospective study was designed and conducted in accordance with the STROBE statement and carried out at the Medical University and University Hospital Innsbruck in patients who underwent colorectal surgery between 1^st^ January 2005 and 31^st^ December 2019. Patients were identified from the prospective, auditable colorectal database (ChiBASE) where all colorectal surgical procedures at the Department of Visceral, Transplant and Thoracic Surgery had been recorded since 2002. Patients were included if a full blood count including hemoglobin and red cell indices were available within 30 days prior to surgery and if follow-up information was available (Fig 1). The information on preoperative iron therapy was not consistently available and could not be included in the study. Type of surgery and clinical diagnosis based on radiological findings and histology were extracted from the colorectal surgery database. Patients not registered with the national health insurance in Austria were excluded because of missing survival data. Further demographic, clinical and biochemical parameters were extracted from the hospital’s electronic health record. For each patient preoperative laboratory parameters including serum iron parameters were recorded for up to 30 days prior to surgery. If laboratory parameters were available at multiple time points during this 30 day interval, the last test result before surgery was considered. This retrospective study was approved by the Ethics committee of the Medical University of Innsbruck (Protocol number 1139/2021). Due to the retrospective nature of the study informed consent did not have to be obtained from study participants, but patients were anonymized after data collection.

Anemia was defined as hemoglobin <120 g/L in females and <130 g/L in males [23]. Iron-deficiency anemia in the cohort of anemic patients was defined as ferritin ≤30 μg/L or ferritin ≤100 μg/L and transferrin saturation ≤ 20%. Anemia of inflammation was defined as ferritin >100 μg/L and transferrin saturation ≤ 20% [24]. Survival status of all patients was determined by a query of the national health database on 29^th^ January 2021. Missing data as potential source of bias were accounted for by list-wise exclusion for patients without available serum iron parameters (Fig 1). Within each cohort missing data were handled by pairwise exclusion.

Statistical analyses were performed using the Software Package for Social Sciences Version 24 (IBM corp. Armonk, NY). Categorical variables are reported as frequencies and percentages and parametric variables as median, 5^th^ and 95^th^ percentile. Differences in medians between groups were tested for statistical significance using Student’s t-test or Mann Whitney U as appropriate. Two by two comparisons of frequencies were tested for significance using the χ^2^-test.

Survival was reported as frequency and percentage of patients alive at discrete time points (30-day and one-year) and further analyzed by the Kaplan-Meier method. Predictors of survival were analyzed by Cox regression analysis. Parameters associated with survival on univariate analysis were included in a multivariate model, where p < 0.05 on univariate analysis was used as threshold for inclusion into the multivariable analysis to reduce the type II error risk.

## Results

**Fig 2:**
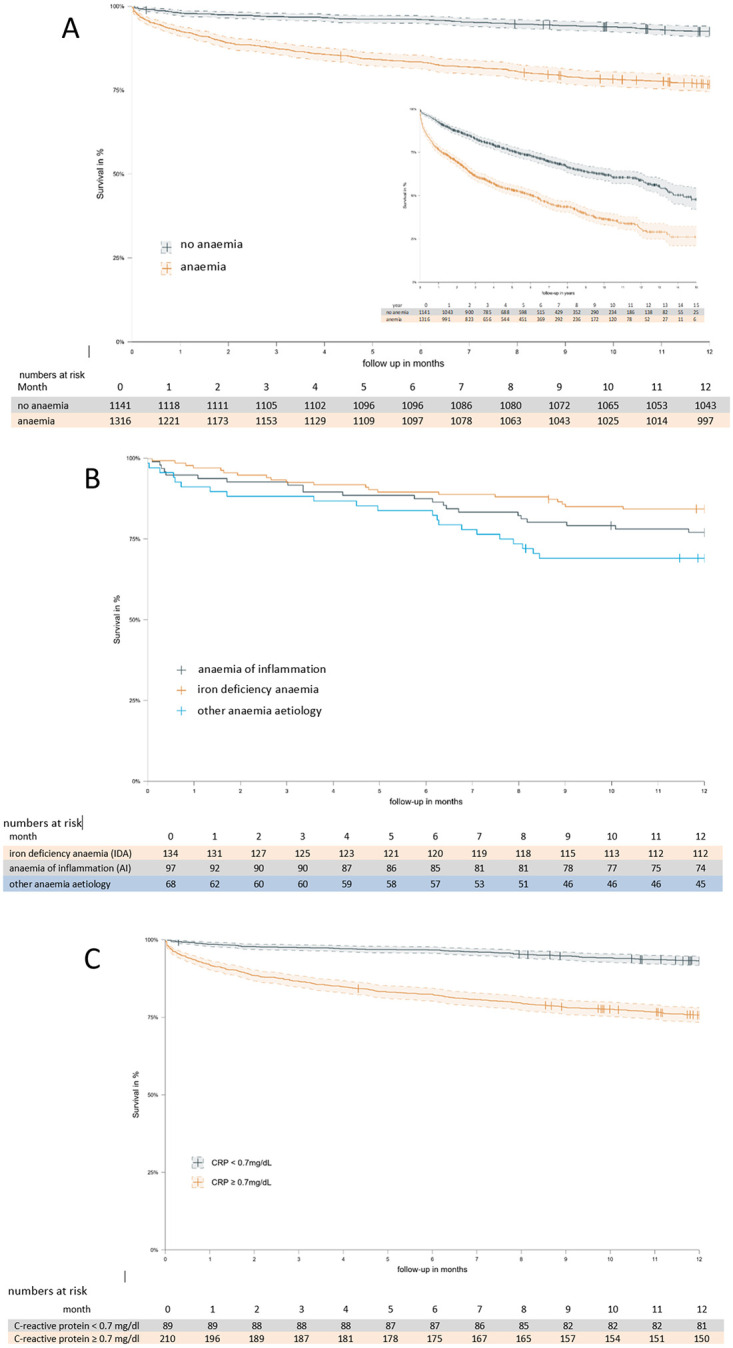
(A) Kaplan-Meier curves and 95% confidence intervals of overall survival during the first postoperative year and the 15 year follow up period (insert) in the cohort of 1316 patients stratified by anemia or no anemia. Differences between groups were analyzed by log-rank test (p < 0.001 for both analyses) (B) Kaplan-Meier curves showing the comparison of overall survival during the first postoperative year in the cohort of 299 patients grouped by anemia etiology as iron-deficiency anemia, anemia of inflammation or ‘other’ anemia etiologies. Differences between groups were analyzed by log-rank test (p = 0.03) (C) Kaplan-Meier curves and 95% confidence intervals showing the comparison of overall survival during the first postoperative year in the cohort of 1305 patients grouped by CRP < 0.7mg/dL or ≥0.7mg/dL (log rank test; p <0.001).

Between January 2005 and December 2019 a total of 3048 colorectal surgical interventions were carried out at the University Hospital of Innsbruck, of whom 2458 could be included (Fig 1). As shown in Table 1, the prevalence of preoperative anemia in this group was 53.5% (1316/2458). When preoperative parameters were compared between patients with anemia and non-anemic patients, patients with anemia were on average significantly older, had higher C-reactive protein (CRP) concentrations, lower transferrin and lower transferrin saturation. Comparing survival in anemic with non-anemic patients, significantly worse outcome in patients with anemia was confirmed at 30 days, at one year (primary endpoint) and during long term follow up of 15 years (log-rank test p<0.05, Fig 2A). Subsequent survival analyses were focused on the first postoperative year, because divergence of survival curves was most prominent during this time period.

**Table 1:** Entire cohort anemia vs. no anemia. (Data are presented as numbers and percentages for frequencies and as mean ± standard deviation for normally distributed parameters and as median (25^th^—75^th^) percentile for not normally distributed parameters).

	n	anemia	n	no anemia	p-value
**n (female/%)**	1316	629 (47.8%)	1141	592 (51.9%)	0.043
**Age (years)**	1316	68.4 (31.4–87.5)	1141	64.2 (31.6–83.6)	**< 0.0001**
**Body mass index (kg/m^2^)**	727	24.0 (17.1–33.6)	886	24.5 (17.5–33.1)	0.075
**Malignancy**	1316	1316 591 (44.9%)	1141	1141 476 (41.7%)	0.111
**Type of surgery**	1316		1141		**< 0.0001**
**Rectum surgery**		343 (26%)		404 (35%)	
**(Procto-)colectomy**		64 (5%)		47 (4%)	
**Hemicolectomy left-sided**		78 (6%)		62 (5%)	
**Hemicolectomy right-sided**		990 (25%)		246 (22%)	
**Partial colectomy (other)**		932 (30%)		284 (25%)	
**Ileocaecal resection**		73 (6%)		73 (6%)	
**Multivisceral resection**		36 (3%)		25 (2%)	
**Creatinine (mg/dL)**	1298	0.88 (0.49–2.47)	1122	0.86 (0.58–1.33)	**0.007**
**Hemoglobin (g/L)**	1316	107 (84–127)	1141	138 (123–162)	**< 0.0001**
**MCH (pg)**	1316	29 (22–33)	1141	30 (27–33)	**< 0.0001**
**MCV (fL)**	1316	86 (71–96)	1141	88 (80–96)	**< 0.0001**
**MCHC (g/L)**	1316	332 (305–354)	1141	343 (324–361)	**< 0.0001**
**C-reactive protein (mg/dL)**	1305	2.70 (0.08–31.82)	1128	0.32 (0.05–14.06)	**< 0.0001**
**Iron (Mmol/L)**	322	5.2 (1.7–23.5)	169	13.6 (3.9–30.1)	**< 0.0001**
**Ferritin (Mg/L)**	299	104 (8–976)	158	81 (18–387)	0.286
**Transferrin (mg/dL)**	305	214 (78–362)	158	248 (152–364)	**< 0.0001**
**Transferrin saturation (%)**	303	11 (3–59)	158	22 (8–49)	**< 0.0001**
**Mean overall survival (dyears)**	1316	3.00 (0.04–11.40)	1141	5.32 (0.62–13.85)	**< 0.0001**
**30-day survival rate**	1221	92.8%	1118	98.1%	**< 0.0001**
**1-year survival rate**	991	76.7%	1043	92.5%	**< 0.0001**

Serum iron parameters were available from 299 of 1316 anemic patients (22.7%). As shown in Fig 1, the prevalence of iron deficiency among patients with anemia and available serum iron parameters was 44.8% (134/299). Patients without iron deficiency were further sub-grouped as AI (serum ferritin >100 μg/L and transferrin saturation ≤ 20%) or other anemia etiology. When the three anemia etiology groups (IDA, AI and other anemia etiology) were compared, no differences in hemoglobin concentrations were found between groups. Further comparison revealed significant differences in median creatinine and CRP concentrations. Red cell indices (MCH, MCV and MCHC) and serum iron parameters were also significantly different among the three groups (Table 1). Mean overall survival of patients with IDA was 8.0 years (95% CI 6.7; 9.3), 7.3 years (95% CI 6.0; 8.5) in anemic patients with inflammation and 5.7 years (95% CI 4.3; 7.0) in the group of anemic patients of other etiology (Fig 2B). When all three groups of patients were compared by Kaplan Meier analysis considering only the first year of follow up, significant differences were found with best outcome among patients with IDA, followed by patients with AI (log rank p = 0.038; Fig 2C). These differences remained significant also after two years of follow up.

**Fig 3:**
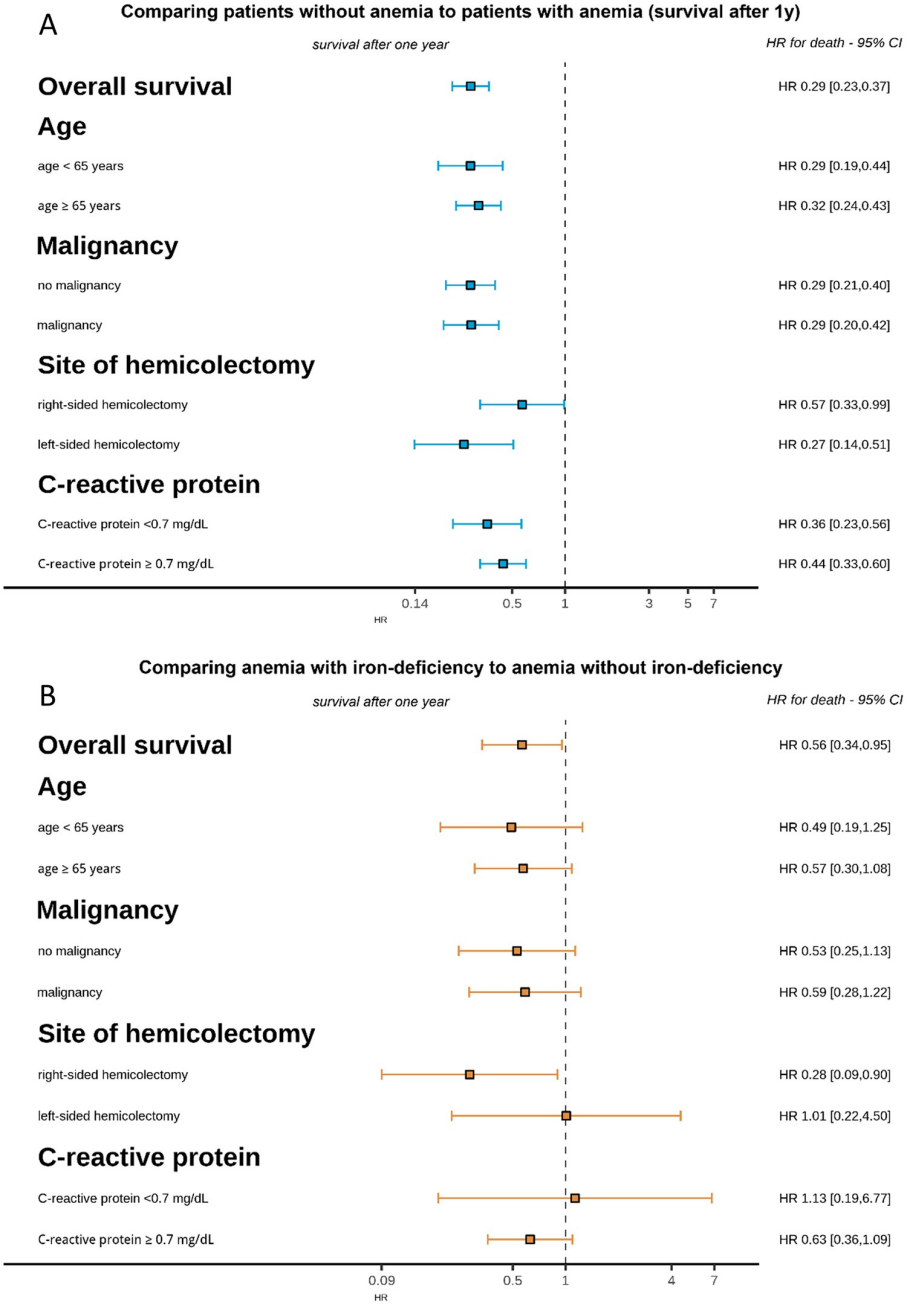
Subgroups analysis comparing hazard ratios and 95% confidence intervals for 1-year survival in indicated patient subgroups (A) with and without anemia and (B) with iron deficiency anemia versus anemia without iron deficiency (corresponding survival analysis is shown in S1 Fig).

To determine if IDA was associated with favorable outcome in different subgroups, hazard ratios for survival during the first postoperative year were analyzed. For this analysis, the group of 1316 patients with preoperative hemoglobin available was stratified by age, malignancy, site of hemicolectomy and CRP. As shown in Fig 3A, the unfavorable association of anemia with outcome was confirmed in all subgroups. When IDA was compared with the combined group of patients with AI and ‘other anemia’ (S1 Fig), lower hazard ratios for death during the first postoperative year did no longer reach statistical significance, but were consistently lower for iron-deficiency anemia patients in most subgroups except for patients undergoing left-sided hemicolectomy and patients with normal CRP (Fig 3B).

When predictors of survival were analyzed in the group of patients with available serum iron parameters, age, hemoglobin, CRP, anemia etiology, ferritin and transferrin were predictors of survival. On multivariate analysis, anemia etiology, younger age and low CRP remained significant independent predictors of improved survival (Table 2). The strong association between CRP and survival was also confirmed by Kaplan Meier analysis, when patients were grouped by normal (<0.7 mg/dL) or elevated CRP (≥0.7 mg/dL) (Fig 2C). Anemia remained a strong negative predictor of survival also when patients were stratified by baseline CRP, but a stronger negative effect of anemia on survival was found in the group of patients with CRP <0.7 mg/dL (Fig 3).

**Table 2:** Characteristics of patients with anemia and serum iron parameters available grouped by anemia etiology. (Data are presented as numbers and percentages for frequencies and as mean ± standard deviation for normally distributed parameters and as median (25^th^—75^th^) percentile for not normally distributed parameters).

	Iron deficiency Anemia	Anemia of inflammation	Other anemia etiologies	
**n (female/%)**	134 (63/49%)	97 (38/39%)	68 (25/37%)	0.150
**Age (years)**	65.6 (26.2–89.1)	62.7 (23.5–85.6)	70.2 (36.7–88.1)	**0.07**
**Body mass index (kg/m^2^)^1^**	22.7 (14.9–31.1)	24.0 (17.2–35.6)	24.0 (17.2–34.6)	0.055
**Malignancy**	71 (53%)	35 (36%)	32 (41%)	**0.038**
**Type of surgery**				**< 0.001**
**Rectum surgery**	22 (16%)	22 (23%)	12 (18%)	
**(Procto-)colectomy**	10 (7%)	8 (8%)	6 (9%)	
**Hemicolectomy left-sided**	3 (2%)	3 (3%)	4 (6%)	
**Hemicolectomy right-sided**	57 (43%)	28 (29%)	20 (29%)	
**Partial colectomy (other)**	26 (19%)	26 (27%)	17 (25%)	
**Ileocaecal resection**	15 (11%)	8 (8%)	3 (4%)	
**Multivisceral resection**	1 (1%)	2 (2%)	6 (9%)	
**Creatinine (mg/dL)**	0.85 (0.49–1.90)	0.92 (0.51–3.24)	1.04 (0.52–4.83)	**0.022**
**Hemoglobin (g/L)**	106 (86–125)	104 (80–128)	109 (75–129)	0.621
**MCH (pg)**	25.1 (20.2–29.8)	28.3 (23.7–32.6)	29.6 (22.6–33.2)	**< 0.001**
**MCV (fL)**	79.1 (66.1–90.5)	84.6 (73.0–95.2)	87.0 (72.9–98.4)	**< 0.001**
**MCHC (g/L)**	319 (297–345)	334 (314–354)	335 (315–359)	**< 0.001**
**C-reactive protein (mg/dL)**	1.49 (0.06–19.06)	6.64 (0.15–33.66)	1.79 (0.07–24.94)	**< 0.001**
**Iron (Mmol/L)**	4.3 (1.7–14.8)	3.8 (1.6–10.6)	14.2 (6.2–66.3)	**< 0.001**
**Ferritin (Mg/L)**	22.5 (5.8–93.3)	292.0 (113.9–940.9)	246.0 (37.9–2489.1)	**< 0.001**
**Transferrin (mg/dL)**	273 (69–389)	164 (154–267)	193 (46–320)	**< 0.001**
**Transferrin saturation (%)**	7 (3–21)	11 (4–19)	32 (21–114)	**< 0.001**
**Mean overall survival (years)**	8.0 (6.7–9.3)	7.3 (6.0–8.5)	5.7 (4.3–7.0)	0.107
**30-day survival rate**	97.0%	94.8%	91.2%	0.105
**1-year survival rate**	84.3%	77.3%	69.1%	**0.036**

Cox regression analysis in the cohort of anemic patients with available serum iron parameters, showed that age, hemoglobin, CRP, ferritin and transferrin were associated with survival in the first postoperative year (Table 3). On multivariate analysis age, CRP, and anemia etiology remained independent associated with survival. This finding further confirms the strong association between inflammation and survival when all patients were included in the analysis (S1 Table).

**Table 3:** Univariate and multivariate Cox regression on 1-year survival in patients with anemia and available serum iron parameters.

	univariate HR (95% CI)	p-value	multivariate HR (95% CI)	p-value
**Gender** ^1^	0.959 (0.584–1.575)	0.870		
**Age (years)**	1.022 (1.006–1.038)	**0.006**	1.033 (1.015–1.052)	**< 0.0001**
**Creatinine**	1.027 (0.831–1.269)	0.807		
**Malignancy** ^2^	1.023 (0.626–1.671)	0.928		
**Hemoglobin**	0.979 (0.962–0.997)	**0.021**	0.981 (0.963–1.000)	0.053
**MCH**	1.002 (0.930–1.079)	0.963		
**MCV**	1.002 (0.972–1.034)	0.881		
**MCHC**	0.998 (0.982–1.014)	0.829		
**C–reactive protein**	1.138 (1.019–1.058)	**< 0.0001**	1.044 (1.022–1.067)	**< 0.0001**
**Anemia etiology** ^3^	1.476 (1.093–1.995)	**0.011**	1.423 (1.041–1.945)	**0.027**
**Iron**	0.979 (0.948–1.012)	0.210		
**Ferritin**^4^, *	1.027 (1.014–1.040)	**< 0.0001**		
**Transferrin** *	0.994 (0.990–0.997)	**< 0.0001**		
**Transferrin saturation**	1.003 (0.993–1.014)	0.543		

^1^(0 = male/1 = female).

^2^(0 = no malignancy/ 1 = malignancy).

^3^(1 = iron deficiency anemia, 2 = anemia of inflammation, 3 = other anemia etiologies).

^4^(per 10 ng/L change)

*Due to the significant association of ferritin and transferrin with anemia etiology, these parameters were not included in the multivariable model.

## Discussion

The association between preoperative anemia and poor postoperative outcome in colorectal surgery has prompted efforts to reduce anemia burden. These strategies are known as PBM and include preoperative measures to prevent, diagnose and treat anemia in order to reduce the use of blood products and ultimately improve outcome [18, 25]. Despite the well-known association between anemia and overall survival, anemia etiology has not been considered in these studies as potential confounder [26, 27]. The main finding of the present study is that among patients with anemia, anemia etiology is an independent predictor of survival, where the strongest effect is found during the first postoperative year.

This finding is based on data from a large retrospective database, where the prevalence of anemia in patients undergoing surgery was 54%. Serum iron parameters were not available for all patients with anemia. In our cohort just under 25% of patients with anemia (299 of 1316) had serum iron parameters determined within 30 days prior to surgery. The cohort was collected before PBM strategies had been implemented at our center. Since preoperative correction of iron deficiency would be feasible in patients with IDA undergoing elective surgery, serum iron parameters should be determined in patients with anemia [18].

For this study, iron deficiency was defined as serum ferritin < 30μg/L or ferritin < 100μg/L and transferrin saturation <20%. Recent data suggest that even a ferritin of < 45μg/L is associated with increased intestinal iron absorption and this higher threshold of serum ferritin should be used for the diagnosis of iron deficiency [11]. The higher ferritin threshold of <100μg/L and TSAT <20% is suggested in guidelines for the management of patients with inflammatory bowel disease and had been previously used in studies on preoperative iron treatment in patients with anemia [10]. A ferritin threshold of <300μg/L and a TSAT < 25% has also been suggested for the definition of IDA [28]. The differences in definitions and thresholds highlight that the diagnosis of iron deficiency in preoperative patients is still arbitrary.

Despite the limitations of serum iron parameters for the diagnosis of iron deficiency, serum iron parameters can also be used to diagnose anemia of inflammation [29]. For the present study a ferritin >100 μg/L and transferrin saturation <20% have been used to categorize preoperative anemia as AI [30]. AI is a common differential diagnosis in surgical patients [30]. The present study shows that patients with AI have worse outcome than patients with IDA, but the remaining group of patients other etiologies of anemia still had worse outcome during the first years postoperatively. This raises the question what these other anemia etiologies were. Data presented in Table 2 shows that this patient group was had significantly higher ager, higher creatinine and significant higher transferrin saturation were found in this group of patients with ‘other’ anemia etiologies. Higher age and impaired renal function are known risk factors for anemia, but creatinine was no significant predictor of survival and anemia etiology and age were both independent predictors of survival on Cox regression analysis [31]. To assess if poor nutritional status could potentially explain anemia in the group of patients with ‘other etiologies’, body mass index was compared and found to be lowest in the group of patients with IDA. The finding that malignant disorders were less prevalent in the ‘other’ anemia etiology group than in patients with iron deficiency suggests that tumor anemia is also unlikely to cause significantly worse outcome in the latter group [32]. Still, inflammation indicated by elevated CRP was associated with poor outcome in patients with anemia and available serum iron parameters as well as in the entire patient cohort, where CRP was available in the majority of patients. The conclusion from these results is that more refined investigations than serum iron parameters and CRP are required to better understand anemia etiology and its association with outcome. Comprehensive assessment of anemia should also include Vitamin B12 and folate, which was only available in a small minority of patients in this study.

The findings from this study support the recommendation that patients undergoing colorectal surgery should be screened for anemia and if anemia is present, further investigations should be carried out to determine its cause. Preoperative correction of IDA would be expected correct the survival disadvantage. In patients with AI, intravenous iron supplementation could also partly improve survival, but in patients with other anemia causes, iron therapy would not be expected to have a beneficial effect on outcome. This notion could explain why a recent metaanalysis on preoperative intravenous iron supplementation, which included the PREVENTT study did not find evidence for a reduction in transfusion rate after intravenous iron. However, in PREVENTT, patients were included based on anemia, without considering the absence of iron deficiency as exclusion criterion [19].

Potential limitations of the present study are that no causes of death were available for the cohort and that serum iron parameters were not determined in all patients. In addition, preoperative iron therapy could have also affected the study results, but this information could not be ascertained in the present study. Causes of death could not be extracted from the national database as the long follow-up time of up to 15 years would make the assessment of death certificates very difficult. Another limitation of the study is the lack of external validation of the results.

In conclusion, the present study confirms that preoperative hemoglobin concentrations are associated with survival and anemia is a major risk factor of poor prognosis in patients undergoing surgery. Beyond the severity of anemia, which determines early postoperative survival, the etiology of anemia appears to be an independent risk factor. The present study results represent real-world outcome data, which could be used for patient selection in future prospective studies on the effect of preoperative iron therapy. As iron deficiency appears to be associated with more favorable short- and long-term outcome than presumed anemia of inflammation, future studies could also stratify patients by the presence of inflammation at baseline.

## Supporting information

S1 Fig Kaplan-Meier curves showing the comparison of overall survival during the first postoperative year in the cohort of 299 patients grouped by anaemia etiology as iron-deficiency anaemia, anaemia without iron deficiency. Differences between groups were analyzed by log-rank test (p = 0.03).
(PDF)

S1 Table Univariate and multivariate Cox regression analysis of 1-year survival in the entire cohort. (NOTE–serum iron parameters were not available in all patients) ^1^(0 = male/1 = female). ^2^(0 = no malignancy/ 1 = malignancy). ^3^(per 10 ng/L change).
(DOCX)

S1 Data (XLSX)
