# Supplementary figures and images for: Impact of preoperative anemia, iron-deficiency and inflammation on survival after colorectal surgery—A retrospective cohort study
Source: PLoS One. 2022 Jul 27;17(7):e0269309. doi: 10.1371/journal.pone.0269309 (PMC9328530; doi:10.1371/journal.pone.0269309)

# Kaplan-Meier-Plots

*iron-deficiency anemia compared to other types of anemia*

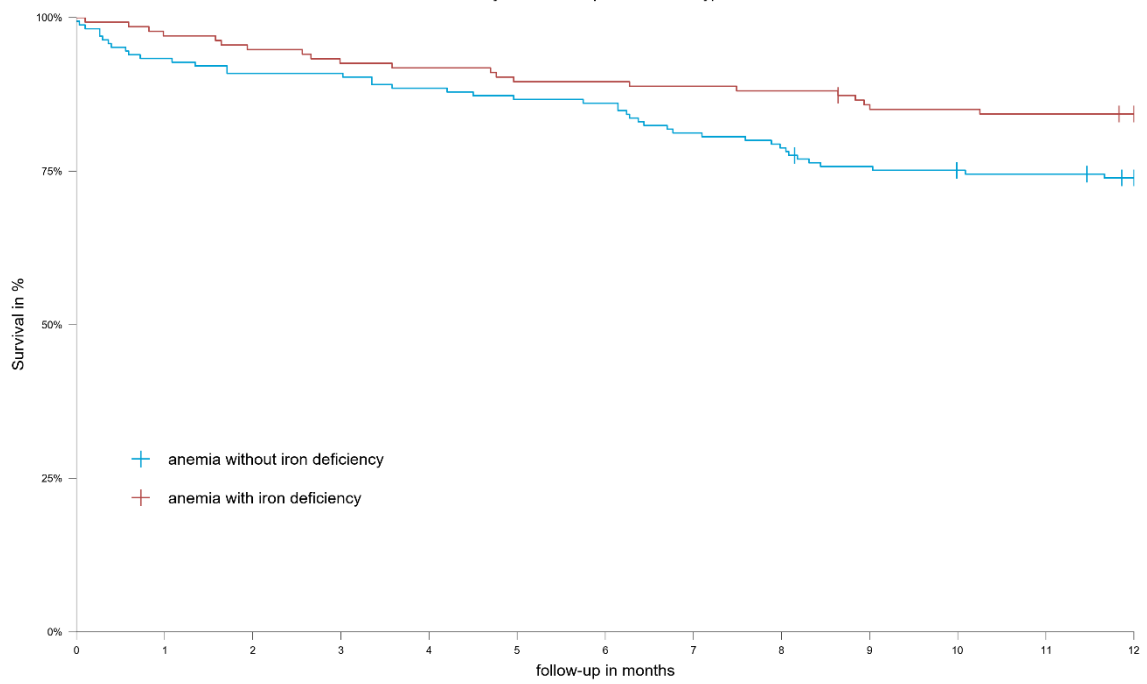

Supplement: S1 Fig — Differences between groups were analyzed by log-rank test (p = 0.03). (PDF) [file pone.0269309.s001.pdf]
