# Supplementary material for: Impact of preoperative anemia, iron-deficiency and inflammation on survival after colorectal surgery—A retrospective cohort study
Source: PLoS One. 2022 Jul 27;17(7):e0269309. doi: 10.1371/journal.pone.0269309 (PMC9328530; doi:10.1371/journal.pone.0269309)
Supplement: S1 Table — (NOTE–serum iron parameters were not available in all patients) 1(0 = male/1 = female). 2(0 = no malignancy/ 1 = malignancy). 3(per 10 ng/L change). (DOCX) [file pone.0269309.s002.docx]

|  | univariate HR  (95% CI) | | p-value | multivariate HR  (95% CI) | p-value |
| --- | --- | --- | --- | --- | --- |
| Gender^1^ | 1.064  (0.873 – 1.298) | 0.537 | |  |  |
| Age (years) | 1.042  (1.034 – 1.050) | **< 0.0001** | | 1.041  (1.024– 1.059) | **< 0.0001** |
| Malignancy^2^ | 1.102  (0.903 – 1.345) | 0.337 | |  |  |
| Haemoglobin | 0.968  (0.963 – 0.973) | **< 0.0001** | | 0.993  (0.977 – 1.009) | 0.389 |
| MCH | 0.957  (0.925 – 0.989) | **0.009** | | 0.884  (0.811 – 0.962) | **0.005** |
| MCV | 0.996  (0.982 – 1.011) | 0.606 | |  |  |
| MCHC | 0.984  (0.977 – 0.990) | **< 0.0001** | |  |  |
| C–reactive protein | 1.041  (1.033 – 1.049) | **< 0.0001** | | 1.047  (1.023 – 1.071) | **< 0.0001** |
| Iron | 0.937  (0.902 – 0.974) | **< 0.0001** | |  |  |
| Ferritin^3, *^ | 1.028  (1.016 – 1.040) | **< 0.0001** | | 1.022  (1.006 – 1.039) | **0.007** |
| Transferrin* | 0.992  (0.989 – 0.995) | **< 0.0001** | | 0.994  (0.991 – 0.998) | **0.002** |
| Transferrin saturation | 0.997  (0.984 – 1.011) | 0.701 | |  |  |

^1^(0=male/1=female). ^2^(0= no malignancy/ 1= malignancy). ^3^(per 10 ng/L change)
